# Mapping breast tissue types by miniature radio-frequency near-field spectroscopy sensor in ex-vivo freshly excised specimens

**DOI:** 10.1186/s12880-016-0160-x

**Published:** 2016-10-10

**Authors:** Zvi Kaufman, Haim Paran, Ilana Haas, Patricia Malinger, Tania Zehavi, Tamar Karni, Izhak Pappo, Judith Sandbank, Judith Diment, Tanir Allweis

**Affiliations:** 1 Breast unit, Meir Medical Center, Kfar Saba, Israel; 2 Department of pathology, Meir Medical Center, Kfar Saba, Israel; 3 Breast unit, Assaf Harofeh Medical Center, Zrifin, Israel; 4 Department of Pathology, Assaf Harofeh Medical Center, Zrifin, Israel; 5 Department of Pathology, Kaplan Medical Center, Rehovot, Israel; 6 Breast Unit, Kaplan Medical Center, Rehovot, Israel; 7 Department of Surgery, Meir Medical Center, Kfar Sava, Israel

**Keywords:** Breast cancer, Lumpectomy, Breast biopsy, Surgical margin, Radio-frequency near-field spectroscopy

## Abstract

**Background:**

Receiving real-time information on tissue properties while performing biopsy procedures has the potential of improving biopsy accuracy. The study goal was to test the ability of a miniature flexible Radio-Frequency (RF) sensor (Dune Medical Devices), designed to be mounted on the surface of surgical tools, in measuring and mapping the various breast tissue types and abnormalities in terms of electrical properties.

**Methods:**

Between January and October 2012, 102 patients undergoing lumpectomy, open-biopsy or mastectomy, in 3 medical centers, were enrolled in this study. The device was applied to freshly excised specimens, with registration between device measurements and histology analysis. Based on histology, the dielectric properties of the various tissue types were derived. Additionally, the ability of the device to differentiate between malignant and non-malignant tissue was assessed.

**Results:**

A total of 4322 measurements from 106 specimens from 102 patients were analyzed. The dielectric properties of 10 tissue types in the low RF-frequency range were measured, showing distinct differences between the various types. Based on the dielectric properties, a score variable was derived, which showed a correlation of 90 % between the RF measurements and the tissue types. Differentiation ability between tissue types was characterized using ROC curve analysis, with AUC of 0.96, and sensitivity and specificity of 90 and 91 % respectively, for tissue feature sizes at or above 0.8 mm.

**Conclusions:**

Using a radio-frequency near-field spectroscopy miniature flexible sensor the dielectric properties of multiple breast tissue types, both normal and abnormal, were evaluated. The results show promise in differentiating between various breast tissue types, and specifically for differentiation between cancer and normal tissues.

## Background

The response of matter to electromagnetic fields is characterized by the material’s dielectric properties: permittivity and conductivity. The conductivity is a measure of the ease with which free electric charges can migrate through the material; the permittivity reflects the extent to which bound charge distributions can be distorted through polarization by an external field.

Differences in dielectric properties between human tissues and specifically between benign and malignant tissue have been studied and are well established [1–3]. Tissue dielectric properties are determined by concentration and mobility of intra and extracellular components, cell size, structure and arrangement, amongst other characteristics. Specifically, the tissue dielectric properties have been extensively studied in breast tissue [4–7], where differences between tissue types, and specifically between normal and malignant were observed over a broad range of frequencies. These properties have been successfully used to differentiate between normal and malignant breast tissue [8, 9] and for intraoperative margin assessment during lumpectomies [10]. Normal breast tissue is heterogeneous, being composed of three different types of tissue (Adipose, Glandular, and Connective), In all the studies to date, the evaluated dielectric properties were based on measurements and comparisons performed on scales larger than the intrinsic scale of tissue heterogeneity within breast tissue. Therefore, the differences observed represent average values of the dielectric properties. Results were generally reported on properties of “normal” and “cancer” types. In some reports [5, 7] an attempt has been made to partition between the three intrinsic tissue types of the normal breast. There has been no reporting on the specific properties of glandular and connective tissue types. Also, cancer of the breast has three major types: Ductal Carcinoma *in Situ* (DCIS), Invasive Ductal Cancer (IDC), and Invasive Lobular Cancer (ILC). The dielectric properties of these types, to date, have not been characterized separately. Additionally, specifically of importance in breast biopsy procedures, the dielectric characteristics of abnormalities in the breast that may progress to cancer, such as: non-malignant proliferative, non-malignant proliferative with atypia, and LCIS, have not yet been characterized.

The burden of breast cancer is high. Approximately 230,480 American women are diagnosed annually, and 39,520 women die from this disease [11]. Global cancer statistics show that breast cancer is the most frequently diagnosed cancer and the leading cause of cancer death among females, accounting for 23 % of total cancer cases and 14 % of cancer deaths [12]. The majority of breast cancers are diagnosed as a result of an abnormal mammogram or ultrasound, and in selected populations abnormal MRI findings. Some lesions are found by the patient or her physician as a palpable mass. Not all abnormal findings diagnosed by the methods mentioned represent cancer. To determine whether a mass in the breast is a suspicious mass or not the BI-RADS System was developed [13]. All patients with a BI-RADS category of 4 and 5 should undergo a biopsy. Those with category 3 should be followed more frequently. A clinically suspicious mass should also be biopsied, regardless of imaging findings, as about 15 % of such lesions can be mammographically occult [14].

Screening mammography is the most common way to diagnose early breast cancer but carries a high rate of recalls (16.3 % at first and 9.3 % at subsequent mammography) [15]. Biopsy is further recommended in 0.6–1 % of all screened women [16, 17]. Millions of women are screened each year, therefore these figures represent a high number of breast biopsies performed each year, emphasizing the need for accurate biopsy.

During the breast biopsy procedure part or all of a suspicious breast tissue growth is sampled and examined for the presence of cancer, most often in a minimally invasive procedure. Current biopsy techniques have several limitations: First, patients diagnosed with Atypical Ductal Hyperplasia (ADH) are routinely sent for an open surgical biopsy, following which 10–25 % of these patients’ diagnosis will be ‘upgraded’ DCIS, which requires a further lumpectomy or mastectomy [18–21]. Second, patients with a diagnosis of DCIS in biopsy will undergo lumpectomy, typically without a sentinel lymph node biopsy. Following the Lumpectomy, about 20 % of these patients’ diagnosis will be upgraded to invasive cancer, requiring a further surgery for node biopsy. Third, studies have shown that up to 10 % of patients endure repeat biopsies [22, 23]. These repeat biopsies reveal carcinoma in up to 25 % of cases [24]. Forth, published data show a 1–7 % false negative rate with current breast core biopsy techniques [25].

The inaccuracies in the biopsy procedure result mainly from the uncertainty in the exact location from which the biopsy sample is taken relative to the image, and from the fact that the features presented on imaging may not be the most abnormal tissue present. Having an *in-situ*, at the needle tip, tissue characterization ability when performing biopsy procedures has the potential to increase the accuracy of the procedure.

In the presented study, we use a miniature, 0.8 mm in diameter, RF sensor to characterize the dielectric properties of different breast tissue types and abnormities. Based on these characteristics, we tested the potential of this type of sensor in differentiating between normal, abnormal, and malignant breast tissue. The sensor has a coaxial opening that results in a fringing electrical field close to the sensor surface. The sensor is manufactured using flexible printed circuit board technology and can be potentially placed on various devices having different geometries, such as open surgical tools as well as minimally invasive ones, like core biopsy needles. A similar device has been already been used for evaluating freshly excised radical prostatectomy specimens [26].

## Material and methods

### Ethics, consent and permissions

The study was performed at 3 sites under institutional review board approval and in accordance with the Declaration of Helsinki. All subjects signed an informed consent.

### General design

Between January to October 2012, subjects undergoing lumpectomy, open biopsy or mastectomy procedure were enrolled. Inclusion criteria specified subjects over 18 years of age.

Tissue measurements were performed on freshly excised breast specimens. Measurements were compared to histological analysis. All medical staff members were blinded to device output. Specimen handling before and after measurements was performed according to routine procedures. As the device was used on ex-vivo specimens in the pathology lab, safety aspects (adverse effects) of the study and device use were monitored only with regards to specimen handling and analysis.

### Device description

The device (Dune Medical Devices Ltd., Israel) used in this study is a near-field Radio-Frequency (RF) spectroscopy-based real-time detection device. It consists of a hand-held, pencil like, probe, connected by cables to a console. The console sends RF waves at several frequencies, transmitted to the tissue through the sensor at the probe’s tip. The frequencies (4–30 MHz) were chosen based on design considerations, and on where differences between breast tissue types are expected to be substantial [5, 6]. The frequency range at which the tissue is probed has no ionizing effects. The amplitude of the transmitted RF field across the sensor opening is low. The voltage across the sensor is ~ 0.1 Volts, much lower than the ionization voltage. The transmitted RF power is ~ 0.1 mW per square mm. Each measurement takes ~ 3 msec. The RF signals are reflected from the tissue through the sensor, and are received by the console. The sensor is designed as a multilayer transmission line structure with a coaxial end opening. It is fabricated from flexible circuit board materials, making the sensor (including the transmission line structure) mechanically flexible and thin. The sensor coaxial end conductive regions are gold–plated. The diameter of the sensor coaxial end is 0.8 mm. The sensor end is attached to the flat tip of the probe. The size of the coaxial sensor is much smaller than the transmitted wavelengths, as the highest frequency of 30Mhz corresponds to a wavelength of 10 m. Under this condition the penetration depth of the field is ~0.078 of the coaxial opening [27]. This translates to a penetration depth of ~0.07 mm.

The sensor has a coaxial opening that results in a fringing electrical field close to the sensors surface, which enables direct calculation of the impedance (dielectric properties - permittivity and conductivity) of the tissue in contact with the sensor. Signals at specific frequencies are transmitted to the sensor. The fringing field interacts with the tissue so that the reflected signals are dependent on the impedance of the tissue close to the sensor. From the reflection amplitude and phase the impedance of the tissue at each frequency measured is extracted using the process described in [28].

### Tissue measurements and processing

Following excision, the fresh specimens were directly delivered to the pathology laboratory; the fresh, non-fixed, specimens were then fully inked and sliced into 5–7 transverse sections, approximately 1 cm thick, in a bread-loaf manner.

**Fig. 1:**
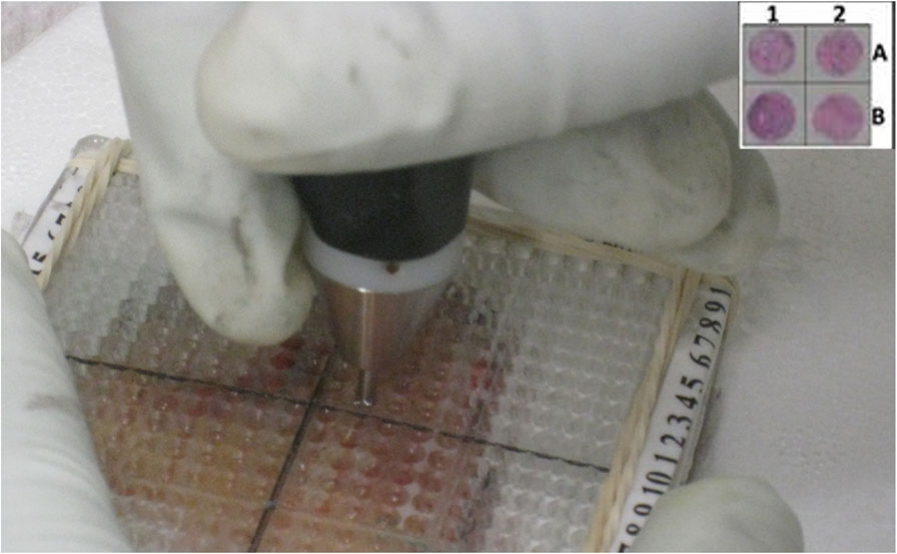
Measurement method of the tissue slices with and apparatus and stencil probe. Inset: histology slide of four measurement sites with their respective stencil coordinates

One or two transverse-cut sections were immediately sampled by the probe. The time between performing the measurements and tissue excision was no longer than 30 min. Specially designed stencils were placed on the slice. These stencils contained a matrix of holes of diameter 3.2 mm, which accommodated the probe’s distal end, enabling it to contact the tissue surface (Fig. 1). The grid spacing was 6 mm. The probe was operated manually to measure all measurement sites of the slice through the stencil (Fig. 1). Good contact between the sensor and tissue was achieved by applying mechanical pressure by hand to the tissue in the stencil holes. Each individual measurement took 1–3 s to complete. Designated software utility helped the user to record the location of the measurements within the stencil. Once a slice was fully measured it was fixed (24–48 h) and further processed for histological analysis. The histological analysis of the slices was performed en-face, without any further sectioning, so that the full surface of the measurement locations was available for histological examination. The relative position of the stencil and the slice was maintained, thus the sampling locations remain fully registered. The Histological slides were prepared for each measured slice. As the diameter of the stencil holes is 3.2 mm and the probe tip diameter is 3.0 mm, it is estimated that the potential registration error between the actual measured site and the analyzed tissue sample was less than 0.1 mm.

### Data analysis

#### Tissue

Each measurement site was histologically analyzed. The tissue composition and feature sizes of the various tissue types present were recorded. The sites were then classified according to their most advanced abnormality within the 1 mm central area and divided into 6 categories [29]: 1) normal and non-malignant non-proliferative abnormal tissue; 2) non-malignant proliferative abnormal tissue; 3) non-malignant proliferative tissue with atypia; 4) LCIS; 5) DCIS and 6) invasive cancer.

#### Measurements

The permittivity and conductivity of each measured tissue site were extracted at all measured frequencies.

#### Dielectric properties

A subset of measurements in which the size of a given tissue type, the feature size, was larger than 0.7 mm was used for assessment of dielectric properties of various tissue types. As the sensor’s size is 0.8 mm, selection of such feature sizes ensures that the sensor came in contact with the specific tissue type being assessed. Analysis was performed by linear regression [30]. Only tissue types presenting in >5 samples were considered. This provided an estimation error of no more than 50 %.

#### Score variable

The same subset of measurements used to determine the dielectric properties of tissues was used in order to derive a single score variable. The score variable was calculated as a linear combination (using linear discriminant analysis) [31] of the dielectric properties, a linear combination that exhibited differentiation between two selected categories, e.g. normal and malignant tissue. The discriminant analysis was performed for each measurement frequency separately. The analysis presented is based on the results for 10 Mhz. The correlation of the score variable values with tissue type categories was characterized by a linear regression analysis.

#### ROC curve analysis

A dichotomous device output (positive/negative) for each measurement was derived by placing a threshold for the score variable value. Measurements with a score value lower than the threshold were defined as being negative, and those above the threshold were defined as being positive. Device indication (positive/negative) of qualified measurements was compared to tissue histology. The threshold levels of the score variable were scanned to generate receiver operating characteristics (ROC) curves. Sensitivity and specificity at the optimal cutoff points (points on the ROC curve closest to the upper left corner of the axes) were extracted. This analysis was repeated for normal vs. all abnormal types (malignant and non-malignant). The score variable of normal vs. malignant tissues was also used to estimate the differentiation ability as a function of the malignant tissue feature size.

## Results

Out of 104 enrolled subjects, 102 were analyzed (their baseline characteristics are presented in Table 1). Two subjects were excluded from the analysis: one due to neo-adjuvant treatment and one specimen was inserted into formaldehyde prior to device use. Four subjects had bilateral surgery, thus 106 specimens were included in the analysis. Device measurements were found to be non-destructive and had no effect on the specimens or on the ability to inspect them histopathologically.

**Table 1:** Patient Cohort

Characteristic	Value
Number of Patients	102
Number of Specimens	106
Age, mean (range)	57 (18–9) years
Procedure	
Lumpectomy	74
Open biopsy	17
Mastectomy	15
Lesion size, mean (range)	1.9 (0.2–13.5) cm
Histology	
IDC	50
ILC	6
DCIS	21
Mixed invasive	6
Other cancer	4
Non-malignant^a^	19
ER/PR^b^	
+/+	64
-/-	7
+/-	9
HER2^c^	
+	24
-	41

^a^5 ADH, 10 Fibroadenoma, 4 Other

^b^9 Undetermined

^c^24 Undetermined

In the 106 specimens included in the analysis 5262 measurements were performed. Altogether 940 measurements (18 %) were not analyzed. 388 sites were not reproduced during slide preparation, i.e. no histology to compare to measurement, and in 119 sites the exact registration between measurement site and histology could not be verified. The remaining 433 sites were disqualified due to predefined criteria related to poor quality of the histology slides (fragmented tissue, torn tissue, uneven tissue surface, color covering sample). Altogether, 4322 measurements were analyzed. The distribution of tissue histology of all qualified tissue measurements is shown in Table 2. Note that the numbers present the most advanced abnormality, according to the established 6 category scale described earlier, within each tissue site analyzed. This was recorded irrespective of size of the abnormality within the sensed area. For example, in some of the sites categorized as malignant some non-malignant abnormalities may be present, as well.

**Table 2:** Distribution of tissue histology

	Tissue type	Number of samples
Malignant	In- Situ	111
	Invasive	390
Abnormal (Non-Malignant)	Hyperplasia	25
	Cyst	41
	Misc. Non Proliferative	63
	Fibroadenoma	210
	Atypia	19
	Misc. Proliferative	10
	LCIS	2
Normal	Adipose	2435
	Connective	777
	Mixture	239

### Dielectric properties

**Fig. 2:**
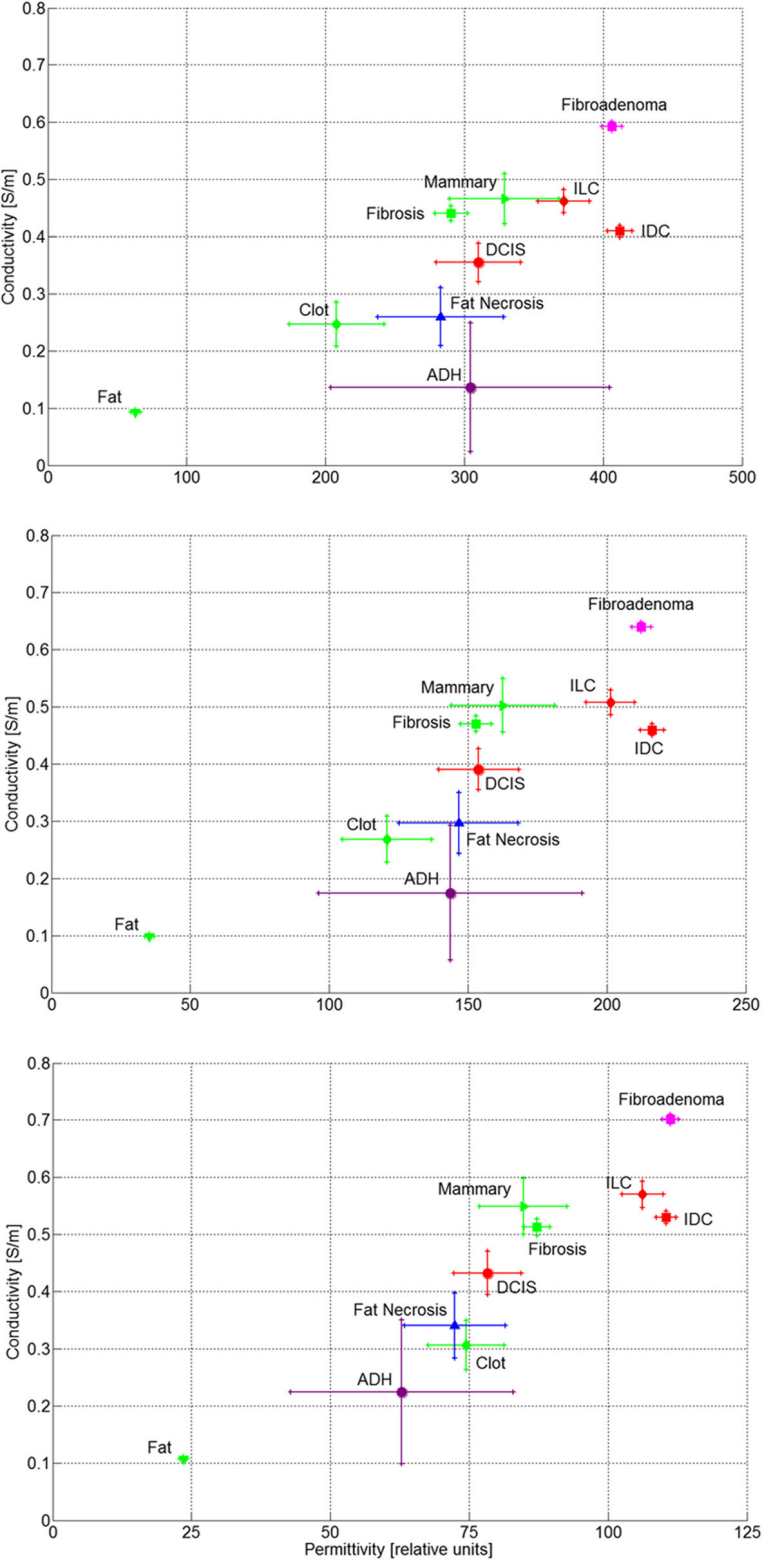
Dielectric properties, evaluated from measurements performed at 4, 10 and 30 MHz (*top to bottom*), of various tissue types

The dielectric properties (conductivity, y-axis, and permittivity, x-axis) of 10 tissue types, as assessed from the data at 4, 10 and 30 MHz, are presented in Fig. 2. The assessment was based on a subset of 1253 tissue measurements which had feature sizes above 0.7 mm of a single tissue type. The dielectric properties were calculated using a linear regression with respect to the relative tissue compositions of these samples. Only tissue types that were present within at least 5 samples were analyzed. The estimation error for each tissue type is also presented. The error depends on the actual spread of values as measured, the feature sizes, and the number of the samples at which a given tissue type is present (e.g. the error for ADH is larger as the number of samples containing ADH was only 7). Additional tissue types present in the breast were either not present in the subset or were very rare, rendering the assessment impractical. The results are similar across the frequency range measured, but the distinction between the various breast tissue types is clearer at 4 and 10 MHz.

### Correlation between tissue types and score variable

**Fig. 3:**
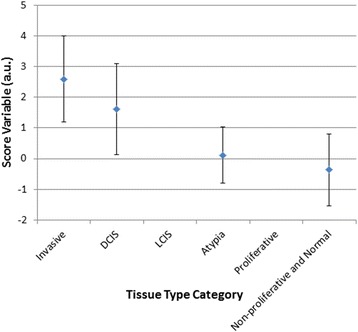
Correlation of score variable (blue diamond denotes the average value and the bars the standard deviation) and tissue type divided into 6 categories

Figure 3 presents the average (and standard deviation) of the score variable (y-axis), derived based on the tissue dielectric properties, vs. the tissue type categories (x-axis). Although tissue were grouped into 6 categories, in the data used (feature size >0.7 mm) for this analysis only 4 categories were present (after excluding Fibroadenoma). LCIS and proliferative lesions were not present in these samples. The more severe the tissue abnormality, progressing from left to right, the higher the score variable, starting at -0.4 (+/- 1.2) for normal and non-proliferative abnormal tissue, up to 2.6 (+/- 1.4) for invasive cancer. The linear regression coefficient between the score variable and the tissue type grouping for the dataset was 0.59 [95 % CI: 0.11–1.07, *P* = 0.033]. The need to identify an abnormality such as Fibroadenoma is usually in young women, where these are the majority of the biopsy findings. In these young women the prevalence of cancer (or atypia) is very low. In women who are biopsied with more “suspicion” for cancer, the more relevant differentiation required does not include Fibroademona. Therefore, Fibroademona presents an “isolated” tissue type, with the background tissue being, most always, normal breast tissue.

### Differentiation between tissue categories

**Fig. 4:**
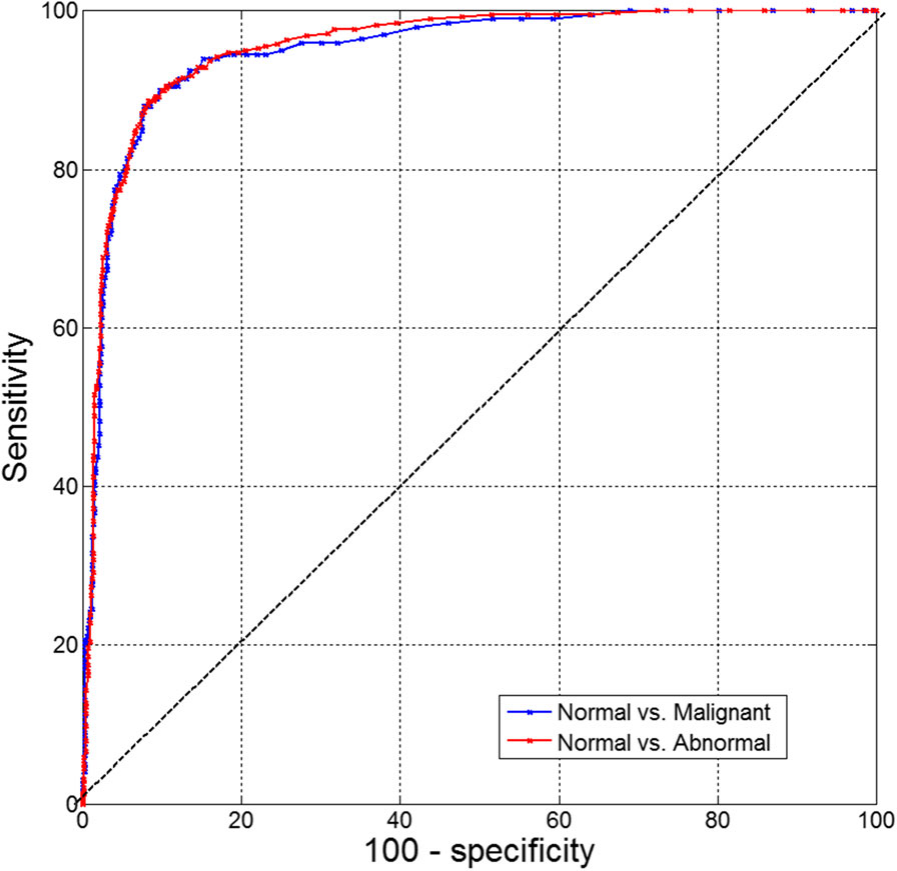
ROC curve analysis of normal vs. malignant measurement sites (*blue*) and normal vs. abnormal measurement sites (*red*) in the subset with feature sizes of >0.7 mm

It is clear from Fig. 2 that various tissue types have different dielectric properties. To further assess whether these differences can serve as a basis for differentiation between various tissue categories, the subset of measurements with feature sizes of >0.7 mm was grouped into binary categories. The resulting ROC curves for two interesting cases, normal vs. malignant (blue line) and normal vs. all abnormal types (red line), are presented in Fig. 4. In both cases the curves follow the left and upper axes, reaching very close to the upper left corner, with the areas under the curve approaching unity; 0.95 (95 % CI: 0.94–0.96) and 0.96 (95 % CI: 0.94–0.97), respectively. The sensitivity and specificity evaluated for each curve at the optimal cut-off point (the point in the curve closest to the upper left corner) are: Sensitivity 90.5 % (95 % CI: 86–94) and Specificity 90.1 (95 % CI: 88–92) for differentiating between normal and malignant tissue, and Sensitivity 88.6 % (95 % CI: 85–92) and Specificity 91.7 (95 % CI: 90–93) for differentiating between normal and all abnormal tissue types, both malignant and non-malignant.

**Fig. 5:**
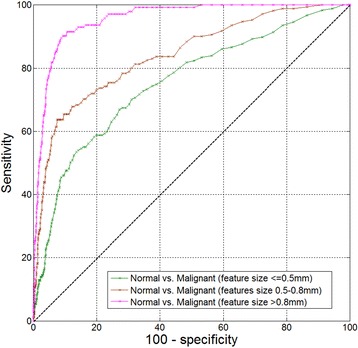
ROC curve analysis of normal vs. malignant measurement sites as function of cancer feature size, for full dataset

In general, it is expected during a biopsy procedure to encounter lesions of ~1 mm or larger. From analysis of SEER (Surveillance, Epidemiology, and End Results program of the National Cancer Institute) data, more than 97 % of the malignant lesions are larger than 3 mm [32]. Still, it is interesting to analyze the separation ability of the device between normal and malignant tissues for various tissue feature sizes. Figure 5 presents 3 ROC curves for 3 feature size categories - above 0.8 mm (the size of the sensor), 0.5–0.8 mm and below 0.5 mm (including any detectable cancer by pathology, even below 0.1 mm). As seen in the figure, the ability to detect cancer (quantified by the areas under the curves, also presented in Table 3) depends, as expected, on the feature size. Table 3 also presents the sensitivity and specificity for each case at the optimal cutoff point of the score variable.

**Table 3:** Differentiation ability for various cancer feature sizes

Feature size	Sensitivity	Specificity	AUG
>0.8 mm	90.2 (95 % CI: 84–95)	90.9 (95 % CI: 90–92)	0.957 (95 % CI: 0.95–0.96)
0.5 to 0.8 mm	73.1 (95 % CI: 66–80)	80.4 (95 % CI: 79–82)	0.84 (95 % CI: 0.83–0.85)
<0.5 mm	66.8 (95 % CI: 60–74)	72.8 (95 % CI: 71–74)	0.75 (95 % CI: 0.74–0.77)

## Discussion

Our results quantify dielectric properties of 10 different tissue types present within the breast, and show that they all have different dielectric properties. These includes the types related to normal breast tissue (Adipose, Glandular, and Connective), and types associated with various abnormalities, including the differentiation between the dielectric properties of the 3 types of cancer types: IDC, ILC, and DCIS. Prior studies [4–7] have demonstrated that, generally, there are differences between normal and cancer tissue within the breast, but have not provided the level of differentiation presented in this work. Additionally, the data on the dielectric properties of pre-malignant types is new. These identified differences served as a basis for constructing a score variable that demonstrated correlation with the degree of abnormality, including pre-malignant phase, of the breast tissue. Additionally, using the dielectric properties and the score variable good differentiation between normal and malignant, or non-malignant abnormal, tissue was established. The configuration of the sensor as a 0.8 mm circular sensor can be thought of as a basic sensing unit for use in breast biopsy procedures, with an array of sensors aligned along the biopsy needle.

The goal of the initial biopsy is to obtain sufficient diagnostic material using the least invasive approach and to avoid surgical excision of benign lesions. CNB offers a definitive histologic diagnosis, avoids inadequate samples and may permit the distinction between invasive versus *in situ* cancer. In most centers, image guided CNB has replaced wire localization and surgical excision as the most common initial biopsy method for nonpalpable abnormalities [33–35]. The accuracy of CNB was shown in a series of 952 consecutive breast CNBs performed at one institution (342 without image guidance, 241 with ultrasound guidance, and 369 using a stereotactic vacuum assisted biopsy (VAB)) [36]. The false-negative rate with 11-gauge VAB was 3 %, compared to 13, 5, and 22 % for non-image guided, surgeon-performed ultrasound-guided, and 14-gauge VAB, respectively. In most of these false negative patients (5–22 %) the reason for the false negative result was due to sampling error, meaning that the biopsy was not taken from the lesion as planned.

The false negative rate can be reduced by using very large needles [37]. An alternative approach could be to keep the needle size relatively small, but add local sensors located at the needle’s tissue collection region that provide *in-situ* information on the tissue about to be sampled. The basic units for these sensors can be sub-millimeter, circular near-field radio-frequency sensing units, as those we have used in this work for characterizing breast tissue properties. These sensors may provide real-time measurements of tissue dielectric properties at the locations of tissue to be biopsied. As the power transmitted by the sensor is very low and the RF radiation is non-ionizing, these type of sensors are well suited for in-vivo use.

As per the current standard of care, imaging will be used to direct the needle to the general location of the suspected abnormality. The in-site sensors will provide indication of the tissue type at the immediate vicinity of the needle and in contact with the sensor, as the sensor is effectively a surface characterization sensor. The penetration depth the 0.8 mm sensor in no more than 0.1 mm. By scanning/moving the needle around the suspected region, the most suspicious tissue abnormality can be identified, and biopsied. The spatial resolution of a sensing device designed using the sensors as the basic building blocks is dictated by the sensor size and by the ability to arrange sensors close together. Arranging 0.8 mm sensors in an array will preserve this resolution, as the sensors can share the same ground plane (the outer conductor of the coaxial aperture).

For a potential set-up for use in a biopsy device configuration, approximately 10 0.8 mm circular sensors will be arranged in a 1D array on the biopsy needle, in a location overlapping the biopsy sampling cavity. As each measurement takes approximately 2.5 msec, a reading from all 10 sensors will take ~ 0.025 s. The full measurement cycle, including displaying the results to the user, will take approximately 0.2 s, thus providing real-time tissue characterization as the needle is progressed through the tissue. Therefore, when the sensors will be integrated with the biopsy needle, it is anticipated that the duration of the biopsy procedure will not be extended.

DCIS is presented mostly as microcalcifications on mammography and diagnosed by stereotactic mammography guided biopsy (mammotomy). The ability of the sensor to distinguish between DCIS and normal breast tissue elements seems promising for using this type of sensor also in mammotomies.

Patients with ADH that were diagnosed on a CNB will be found to have in up to 25 % DCIS or even IDC present at open biopsy. Therefore patients with ADH on CNB are routinely sent for an open surgical biopsy [18–21]. A more accurate CNB can reduce the number of unnecessary open biopsies in these patients. The ability of the sensor to differentiate between normal breast tissue, ADH and DCIS, can potentially improve the accuracy of CNB and reduce the number of open biopsies.

The diagnostic capability of the sensor for differentiating between malignant and normal tissue is high, with a Sensitivity of 90.5 % and Specificity of 90.1 %. This is also reflected by the ROC curves, with an area under the curve of 0.95. The ability of the sensor to differentiate any abnormal tissue from normal tissue is also high, Sensitivity 88.6 % and Specificity 91.7 %. With the current false-negative rates of CNB, this level of sensitivity has the potential of reducing the false negative rates in biopsy procedures to below 1 %.

The detection sensitivity of the sensor is dependent on the feature size of the malignant tissue. A 90.2 % Sensitivity features of at least 0.8 m in size, down to 66.8 % sensitivity for features smaller than 0.5 mm. It is anticipated that most biopsied malignancies will be of at least 1 mm in size, as, based on final pathology, most all of the malignant lesions are found to be larger than 3 mm.

There are some limitations to this type of sensor design. The manufacturing process of the sensor has to account for potential chemical modifications (over time) of the senor face, which can affect the reflected signals. Arranging sensors in a tightly packed array provides an additional challenge in isolating the electrical response of the individual sensors from their neighbors. The tissue has to be in direct contact with the sensor. Therefore, a practical device will need good attachment to substrate, as any (air) voids between the sensor and the tissue will skew the impedance measurement results. The very low penetration depth may limit the scope of applications in which this type of sensor will provide benefit. The sensor is gold-plated, with gold known not to be durable with regards to mechanical abrasion. In a biopsy procedure each sensor would be used for a limited duration, typically no more than a few minutes. Also, breast tissue is soft, and therefore it is not expected that the sensor structure will be mechanically effected during use.

## Conclusion

By use of a miniature flexible radio-frequency near-field spectroscopy sensor the dielectric properties of 10 tissue types present within the breast were quantified, showing distinct differences between the all these types. Using these differences a good differentiating was achieved between breast tissue states, specifically between cancerous and normal tissue. The sensor’s dimensions and design may enable the use of such sensors in minimally invasive procedures, including breast biopsy.

## Abbreviations

ADH: Atypical Ductal Hyperplasia
CNB: Core Needle Biopsy
DCIS: Ductal Carcinoma *In Situ*
RF: Radio Frequency
ROC: Receiver Operating Characteristics
VAB: Vacuum assisted Biopsy
